# Life History Traits of *Mythimana loreyi* (Lepidoptera: Noctuidae) on Two Transformation Events (DBN9936 and Ruifeng 125)

**DOI:** 10.3390/toxins18100420

**Published:** 2026-09-29

**Authors:** Guoping Li, Lingli Meng, Siyuan Feng, Pengjuan Cui, Gemei Liang, Kongming Wu, Qiuju Qin, Hongqiang Feng

**Affiliations:** 1 Xinxiang Observation and Research Station, Ministry of Agriculture and Rural Affairs, Key Laboratory of Integrated Crop Pests Management on Crops in Southern Region of North China, Ministry of Agriculture and Rural Affairs, Henan Key Laboratory of Agricultural Pest Monitoring and Control, No. 0 Entomological Radar Field Scientific Observation and Research Station of Henan Province, Institute of Plant Protection, Henan Academy of Agricultural Sciences, Zhengzhou 450002, China; 2 College of Plant Protection, Hebei Agricultural University, Baoding 071033, China; 3 Seed Industry Development Center of Henan Province, Zhengzhou 450046, China; 4 State Key Laboratory for Biology of Plant Diseases and Insect Pests, Institute of Plant Protection, Chinese Academy of Agricultural Sciences, Beijing 100193, China

**Keywords:** transgenic insect-resistant maize, *Mythimna loreyi*, sublethal effects, life history traits

## Abstract

*Mythimna* (=*Leucania*) *loreyi* (Lepidoptera: Noctuidae) is an important pest of summer maize (*Zea mays*) in China’s Huang–Huai–Hai region. The approved transformation events DBN9936 (Cry1Ab) and Ruifeng 125 (Cry1Ab/Cry2Aj) were primarily developed to target *Ostrinia furnacalis*, and their efficacy against other lepidoptera pests, such as *M. loreyi*, remains largely unknown. Using age-stage two-sex life tables under controlled conditions, we quantified lethal and sublethal effects on these transformation events on first- to third-instar *M. loreyi* larvae fed V6-V8 leaves, and further evaluated how host plant movement scenarios affected larval development. Within a 7-day exposure period, both transformation events exerted significant lethal effects on *M. loreyi*. Corrected mortalities for first- to third-instar larvae were 93.43%, 17.33%, and 8.61% for DBN9936, and 44.33%, 8.53%, and 3.34% for Ruifeng 125, respectively. Individuals that survived on Bt maize exhibited significant sublethal effects, including prolonged larval development (ΔT = 8.12~9.93 d), reduced pupation rates (decreased by 69.41~100.00%), lower pupal mass (reduced by 31.14~33.64%), and decreased fecundity (by 177.86~262.96 eggs/female). Life table analysis revealed that both transformation events suppressed *M. loreyi* population growth in an instar-dependent manner. First-instar larvae failed to survive on either Bt transformation event. For second-instar larvae, DBN9936 remained completely lethal, while Ruifeng 125 allowed minimal survival (*R*_0_ = 7.51, relative fitness = 0.03). For third-instar larvae, partial survival occurred on both Bt events (DBN9936: *R*_0_ = 21.68, relative fitness = 0.09; Ruifeng 125: *R*_0_ = 32.34, relative fitness = 0.14), with *r_m_* values (0.06–0.07), lower than those on conventional controls (0.14–0.16). Host plant movement scenarios also affected *M. loreyi* survival and mass gain in an age-dependent manner. Brief early exposure to Bt maize did not reduce survival, but significantly suppressed body mass, whereas continuous Bt exposure caused drastic mortality in young larvae (2.08–8.33% survival). Older larvae tolerated Bt maize without survival loss, yet still exhibited significantly reduced body mass (*p* < 0.05). These findings demonstrate that DBN9936 and Ruifeng 125 exert substantial significant lethal and sublethal effects on *M. loreyi*, but the ability of older larvae to survive on Bt maize, combined with potential larval movement between Bt and non-Bt plants may facilitate resistance evolution.

## 1. Introduction

*Mythimna* (=*Leucania*) *loreyi* (Duponchel) is a widely distributed, polyphagous, and migratory pest that poses a significant threat to cereal production in China [1,2]. Throughout the 20th century, *M. loreyi* mainly damaged rice and maize in southern China, with fewer reports of damage to sugarcane and wheat, and it rarely caused outbreaks [3,4]. Since the beginning of the 21st century, influenced by climate change, adjustments in cropping structure, and shifts in crop varieties, *M. loreyi* has gradually become one of the major pests of maize in northern China. From 2015 to 2020, infestations of *M. loreyi* on maize were reported in multiple provinces in the Huang–Huai–Hai summer maize planting region, including Anhui, Henan, Hebei, Shandong, and Shaanxi, with some areas experiencing nearly complete consumption of maize seedlings [5,6,7]. In 2020, it was classified as a Category I crop disease and pest in China. Current control strategies rely heavily on chemical insecticides, the prolonged use of which not only contributes to environmental pollution, but also accelerates the development of insecticide resistance [8,9].

Advances in agricultural biotechnology, particularly transgenic technology, offer new approaches for enhancing maize resistance. Since commercialization in 1996, transgenic insect-resistant maize has been widely deployed and serves as an important component of maize integrated pest management (IPM) systems [10]. It exhibits strong efficacy against major target pests, such as the European corn borer (*Ostrinia nubilalis* Hübner) and the southwestern corn borer (*Diatraea grandiosella* Dyar) [11,12]. The long-term sustainability of Bt crops depends critically on the effective implementation of the high-dose/refuge strategy, which requires Bt protein expression levels sufficient to kill ≥95% of heterozygous (RS) individuals and 100% of homozygous susceptible (SS) target pests, thereby eliminating the recessive resistance (R) alleles [13,14,15]. This strategy has successfully delayed the evolution of resistance to Bt crops and Bt cotton in the major lepidopteran pests for over 25 years [16,17,18]. Crucially, when *Bt* expression falls below high-dose thresholds, sublethal exposure enables RS heterozygote survival. This facilitates R allele persistence in pest populations and compromises refugia efficacy [19,20]. For example, Cry1Ab-expressing maize primarily targets *O. nubilalis* and *D. grandiosella*, against which it demonstrates high efficacy [18], however, this trait expresses a moderate-dose phenotype for *Helicoverpa zea* (Boddie), with reported field efficacy of 75–80%, and this sublethal exposure enables resistant *H. zea* genotypes to survive and complete development on *Bt* maize, thereby accelerating resistance evolution or altering population dynamics [18,21].

The two transgenic maize events, DBN9936 (Cry1Ab) and Ruifeng 125 (Cry1Ab/Cry2Aj), which have obtained biosafety certificates in China, show high efficacy against highly susceptible pests, such as the Asian corn borer (*Ostrinia furnacalis* Guenée) and the yellow peach moth (*Conogethes punctiferalis* Guenée). However, both DBN9936 and Ruifeng 125 fail to meet high-dose criteria against *M. loreyi*, indicating a “non-high-dose” exposure risk [22]. Sublethal dose exposure can affect key life history traits, including development and reproduction, thereby altering population dynamics and posing potential ecological risks. Systematic studies on the effects of these Bt maize events on the growth and development of *M. loreyi* remain limited. Therefore, this study aimed to evaluate the lethal and sublethal effects of two non-high-dose transformation events, DBN9936 and Ruifeng 125, on *M. loreyi* through laboratory bioassays, analyzing key life history parameters such as larval development, survival, pupation, and reproductive performance. To the best of our knowledge, this is the first study to systematically quantify the instar-dependent lethal and sublethal effects of the first two Bt maize transformation events granted biosafety certificates in China on *M. loreyi* using age-stage two-sex life tables. By incorporating host plant movement scenarios, this work provides population-level insights into the ecological risks and resistance evolution potential of *M. loreyi* under non-high-dose Bt exposure. These findings are expected to provide a scientific basis for the environmental risk assessment of Bt maize and the development of integrated management strategies for this pest.

## 2. Results

### 2.1. Mortality of M. loreyi on Transgenic Bt Maize

**Figure 1:**
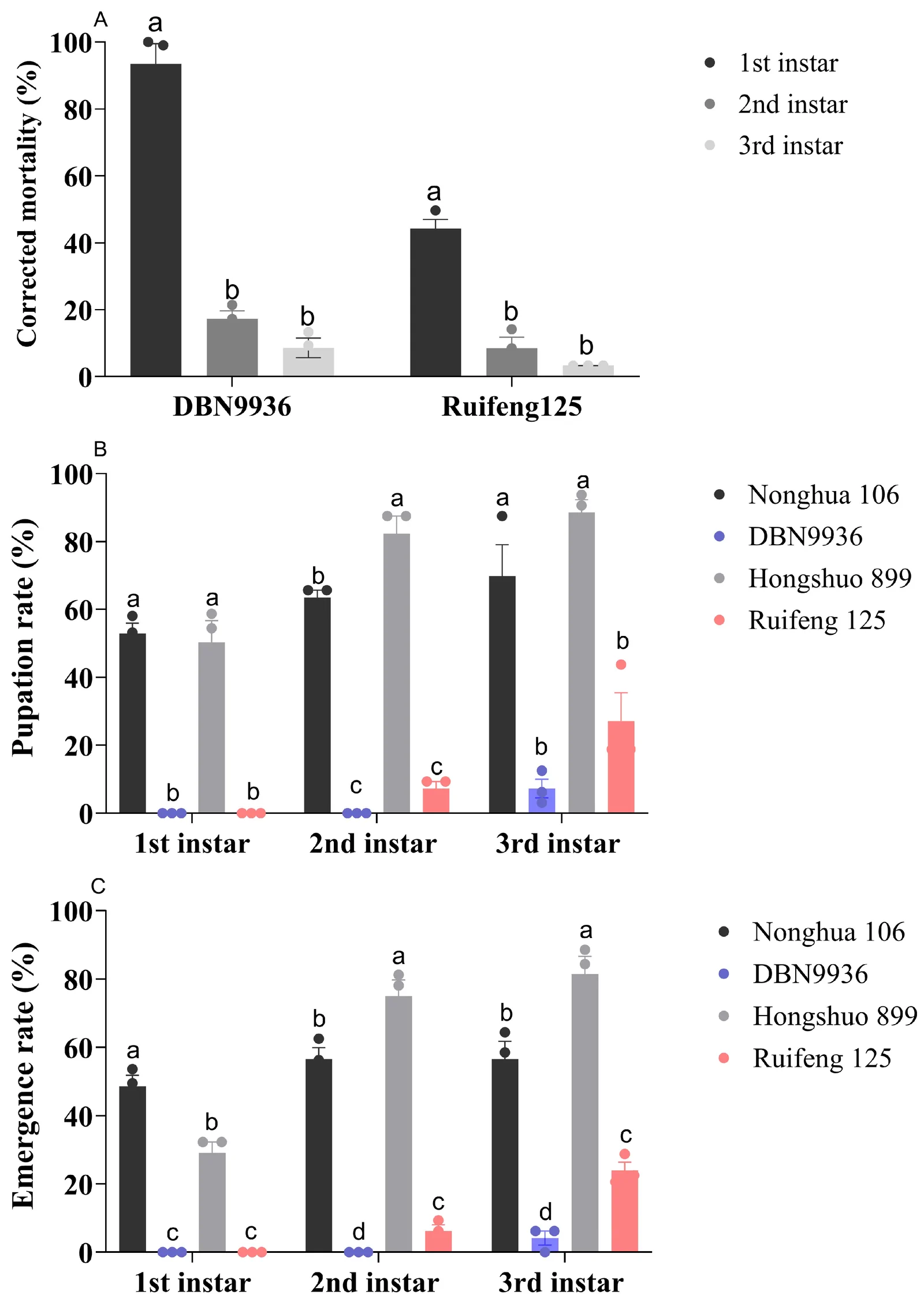
Lethal effects on first- to third-instar larvae of *M. loreyi* fed on DBN9936 (Cry1Ab), Ruifeng 125 (Cry1Ab/Cry2Aj) at the V6-V8 growth stage relative to isogenic negative controls. Bars labeled with different lowercase letters indicate significant differences among treatments (±SE, GLMMs, *p* < 0.05). In panel (**A**), letters compare corrected mortality among different larval instars within the same transformation event; in panels (**B**,**C**), letters compare pupation and emergence rates among the four maize varieties within the same larval instar: (**A**) 7-day corrected mortality; (**B**) pupation rate; (**C**) emergence rate.

Generalized linear mixed models (GLMMs) revealed that maize variety (χ^2^ = 333.21, *p* < 0.001), larval instar (χ^2^ = 1032.03, *p* < 0.001), and their interaction (χ^2^ = 151.96, *p* < 0.001) significantly affected larval mortality. Corrected 7-day mortality rates for *M. loreyi* larvae fed V6-V8-stage transgenic maize are presented in Figure 1. For *M. loreyi* (Figure 1A), mortality reached 93.43 ± 1.63% (first instar), 17.33 ± 4.05% (second instar), and 8.61 ± 0.67% (third instar) on DBN9936, compared to 44.33 ± 2.40%, 8.53 ± 1.33%, and 3.34 ± 0.19% on Ruifeng 125. These results demonstrate significant differences in the susceptibility of *M. loreyi* to Bt leaves, influenced by both the transformation event and larval instar.

Maize variety (χ^2^ = 74.40, *p* < 0.001) and variety × instar interaction (χ^2^ = 91.22, *p* < 0.001) exerted significant effects on pupation rate, whereas the main effect of instar was undetectable (χ^2^ = 0, *p* = 1.00), probably due to complete separation originating from zero pupation in several Bt maize treatments. First-instar larvae of *M. loreyi* exhibiting complete pupation failure (0%) occurred on both Bt maize varieties (Figure 1B). Similarly, second-instar larvae showed no pupation (0%) on DBN9936 (Cry1Ab), while demonstrating minimal pupation (7.29%) on Ruifeng 125 (Cry1Ab/Cry2Aj)—significantly lower than the 82.29% observed on non-Bt Hongshuo 899 (*p* < 0.001). Compared to non-Bt controls (69.79–88.54%), third-instar larvae demonstrated partial but substantially reduced pupation rates on Bt varieties (7.29% on DBN9936; 27.08% on Ruifeng 125) (*p* < 0.001), confirming instar-dependent survival patterns.

Emergence rate was significantly affected by maize variety (χ^2^ = 29.46, *p* < 0.001) and the variety × instar interaction (χ^2^ = 189.38, *p* < 0.001). No pupation occurred in three treatments (both Bt varieties for first instar and DBN9936 for second instar). For the estimable combinations, emergence rate ranged from 4.12% to 81.55% and were significantly lower on Bt maize than on their non-Bt counterparts. For instance, at the third instar, emergence rates were markedly reduced on DBN9936 (4.12%) and Ruifeng 125 (23.83%) relative to Hongshuo 899 (81.55%) and Nonghua 106 (56.64%) (Figure 1C; all *p* < 0.001). These results indicate that, among successfully pupated larvae, adult emergence was significantly reduced on Bt maize in an instar-dependent manner.

### 2.2. Larval–Pupal Development and Pupal Weight of M. loreyi on Bt and Non-Bt Maize

**Figure 2:**
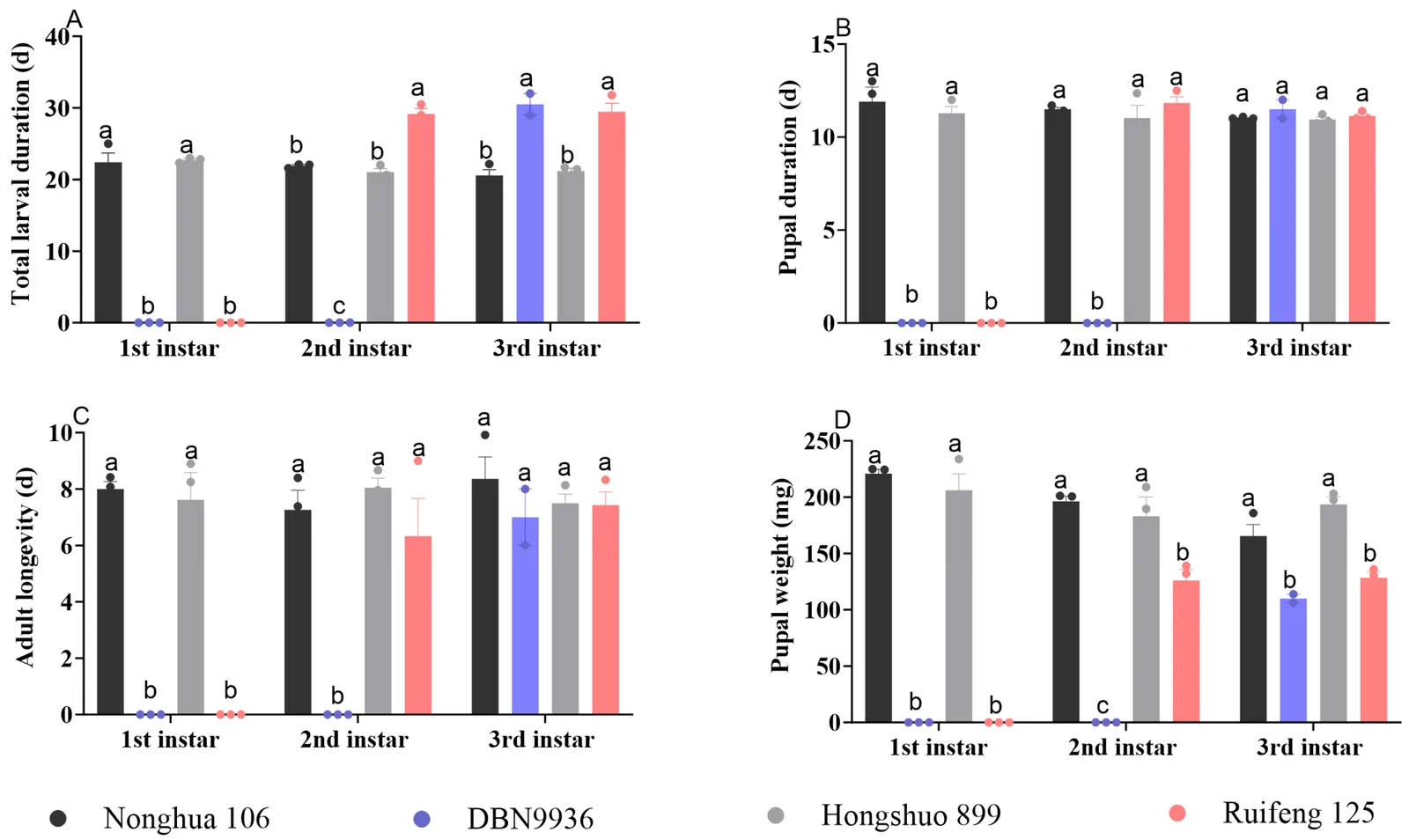
Durations of developmental stages and pupal weight of *M. loreyi* fed on DBN9936 corn (Cry1Ab), Ruifeng 125 corn (Cry1Ab/Cry2Aj) and their isogenic negative controls. Bars labeled with different lowercase letters indicate significant differences among the four maize varieties (±SE, Tukey’s HSD test, *p* < 0.05). (**A**) Total larval duration; (**B**) pupal duration; (**C**) adult longevity; (**D**) pupal weight.

Multifactorial ANOVA revealed significant main effects of maize variety (*F*_3, 23_ = 356.76; *p* < 0.001), larval instar (*F*_2, 23_ = 488.72; *p* < 0.001) and variety × instar interaction (*F*_6, 23_ = 254.60; *p* < 0.001), for total larval duration of *M. loreyi*. Second-instar larvae reared on Ruifeng 125 (Cry1Ab/Cry2Aj) exhibited prolonged larval duration (29.17 ± 0.73 d) compared to Hongshuo 899 (21.05 ± 0.49 d) (*p* < 0.001; Figure 2A). Third-instar larvae on Bt maize DBN9936 (Cry1Ab) and Ruifeng 125 showed significantly extended durations (30.50 ± 1.50 d and 29.46 ± 1.17 d, respectively) versus non-Bt controls Nonghua 106 (20.57 ± 0.80 d) and Hongshuo 899 (21.18 ± 0.40 d) (*p* < 0.001; Figure 2A).

Maize variety, larval instar, and their interaction significantly affected both pupal duration (variety: *F*_3, 23_ = 295.62, larval instar: *F*_2, 23_ = 164.68; interaction: *F*_6, 23_ = 107.71; all *p* < 0.001) and adult longevity (variety: *F*_3, 23_ = 47.83; larval instar: *F*_2, 23_ = 26.12; interaction: *F*_6, 23_ = 15.05; all *p* < 0.001). Nevertheless, pupal duration (10.94–11.91 d) and adult longevity (6.33–8.36 d) of larvae that completed their life cycle did not differ between Bt and non-Bt maize (Figure 2B,C; *p* > 0.05).

Pupal weight was significantly influenced by maize variety (*F*_3, 23_ = 213.57; *p* < 0.001), larval instar (*F*_2, 23_ = 42.43; *p* < 0.001) and their interaction (*F*_6, 23_ = 37.32; *p* < 0.001). Second-instar larvae on Ruifeng 125 produced lighter pupae (126.05 ± 9.76 mg) than those on Hongshuo 899 (183.06 ± 17.16 mg) (*p* < 0.001; Figure 2D). Third-instar larvae on DBN9936 (110.05 ± 3.85 mg) and Ruifeng 125 (128.50 ± 5.14 mg) exhibited reduced pupal weights compared to non-Bt maize Nonghua 106 (165.57 ± 10.19 mg) and Hongshuo 899 (193.64 ± 6.93 mg) (*p* < 0.001).

### 2.3. Larva–Adult Survivorship of M. loreyi Fed Transgenic Bt vs. Non-Bt Maize

**Figure 3:**
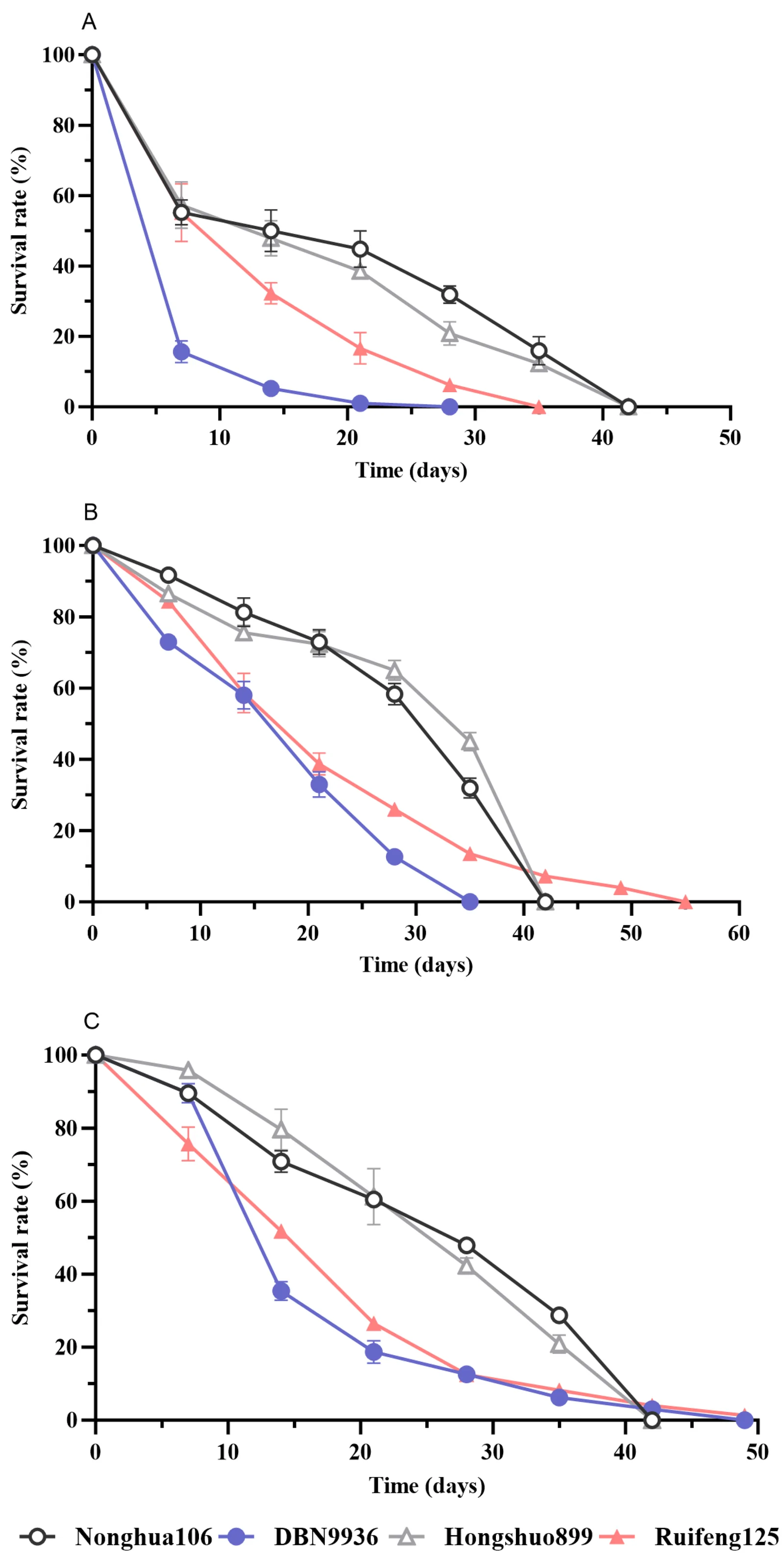
Survival rate from first- to third-instar larvae to adults (complete life cycle) of *M. loreyi* fed on DBN9936 (Cry1Ab), Ruifeng 125 (Cry1Ab/Cry2Aj), and their isogenic negative controls: (**A**) 1st instar; (**B**) 2nd instar; (**C**) 3rd instar.

Larva-to-adult survival of *M. loreyi* is presented in Figure 3. Although first-instar larvae could survive for approximately 30 days on both Bt transformation events, they exhibited complete developmental failure (0% larva-to-adult survival) (Figure 3A). Second-instar larvae failed to pupate on DBN9936 (Cry1Ab), while those on Ruifeng 125 (Cry1Ab/Cry2Aj) showed significantly prolonged developmental duration (55.04 ± 0.92 d vs. 42.13 ± 0.92 d on Hongshuo 899; *F* = 11.46; *df* = 2, 8; *p* < 0.05; Figure 3B). Third-instar larvae reared on DBN9936 (48.50 ± 0.71 d) and Ruifeng 125 (48.03 ± 1.84 d) demonstrated significantly extended development compared to non-Bt controls (Nonghua 106: 41.97 ± 1.62 d; Hongshuo 899: 42.21 ± 1.38 d; *F* = 27.81; *df* = 3, 11; *p* < 0.001; Figure 3C). Both Bt events induced significant developmental delays, extending maturation by 8–9 days on average.

### 2.4. Reproductive Performance of M. loreyi on Bt Maize: Percentage of Females, Fecundity, and Fertility

**Figure 4:**
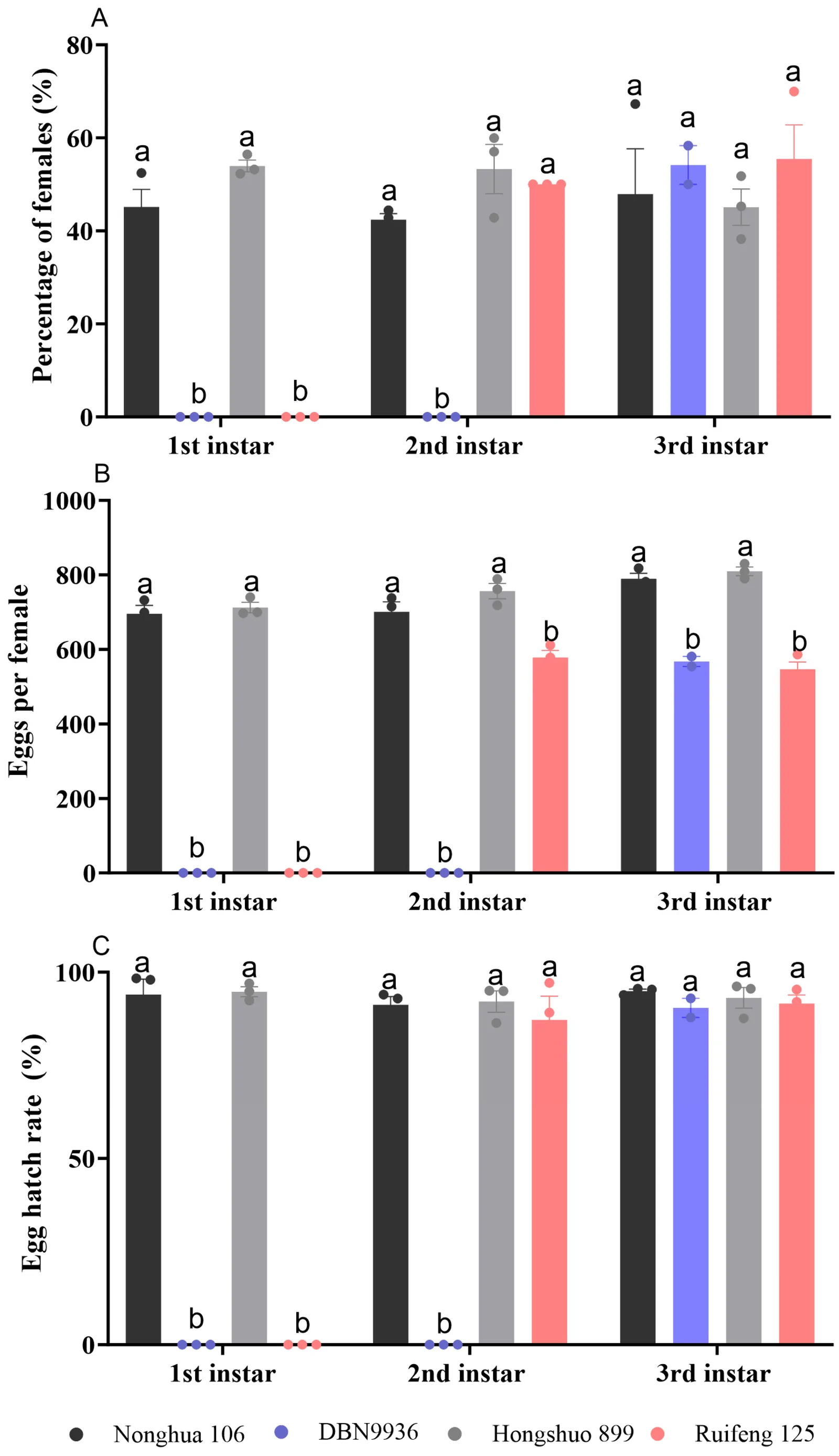
Reproductive performance of *M. loreyi* fed on DBN9936 (Cry1Ab), Ruifeng 125 (Cry1Ab/Cry2Aj), and their isogenic negative controls. Bars represent mean values ± standard error (SE); different lowercase letters denote significant differences among treatments (*p* < 0.05). Panels (**A**,**C**) were analyzed using generalized linear mixed models (GLMMs), whereas panel (**B**) was analyzed by Tukey’s HSD test. (**A**) Percentage of females; (**B**) eggs per female; (**C**) egg hatch rate. Life history traits related to reproduction were measured on surviving individuals only.

Owing to the incomplete factorial design with missing cultivar–instar combinations, full main and interaction effects could not be estimated, and comparisons were performed only for treatment combinations with available observations. Across the 26 valid combinations, 1074 females and 1125 males were recorded, yielding an overall percentage of females of 0.488, close to a 1:1 sex ratio. Estimated percentage of females ranged from 42.49% to 54.12%, with all 95% confidence intervals encompassing 50.00% (the expected value under a 1:1 sex ratio). These results indicate that the tested cultivars and instars did not induce obvious shifts in percentage of females among individuals that completed their full life cycle (Figure 4A).

Fecundity (eggs per female) was significantly influenced by maize variety (*F*_3, 23_ = 635.69; *p* < 0.001), larval instar (*F*_2, 23_ = 309.80; *p* < 0.001), and their interaction (*F*_6, 23_ = 131.68; *p* < 0.001). Second-instar larvae reared on Ruifeng 125 (Cry1Ab/Cry2Aj) exhibited significantly reduced fecundity (578.75 ± 32.70 eggs/female) compared to Hongshuo 899 (756.61 ± 35.40 eggs/female; *p* < 0.001; Figure 4B). Third-instar larvae on Bt maize showed substantially lower fecundity: DBN9936 (Cry1Ab) at 567.64 ± 19.14 eggs/female and Ruifeng 125 at 546.75 ± 34.50 eggs/female versus non-Bt controls Nonghua 106 (789.61 ± 25.43 eggs/female) and Hongshuo 899 (809.71 ± 19.82 eggs/female; *p* < 0.001; Figure 4B).

Because some cultivar–instar combinations were absent, the effects of cultivar, instar, and their interaction could not be evaluated separately. Statistical comparisons were therefore restricted to the treatment combinations with available observations. Estimated hatching probabilities ranged from 89.45% to 95.16%, with overlapping 95% confidence intervals across treatments (Figure 4C). These results indicate that the tested cultivars and instars exerted no significant effects on hatching rate of individuals that completed their full life cycle.

### 2.5. Life History Parameters of M. loreyi on Bt and Non-Bt Maize

*M. loreyi* larvae reared on Bt maize varieties DBN9936 (Cry1Ab) and Ruifeng 125 (Cry1Ab/Cry2Aj) exhibited severe reductions in population fitness parameters compared to non-Bt isogenic controls. Among cohorts with surviving individuals, net reproductive rates (*R*_0_) plummeted by 86.10–96.57% across instars and varieties, with Ruifeng 125-fed second instars showing *R*_0_ = 7.51 (vs. 219.22 in Hongshuo 899 controls). Concurrently, mean generation time (*T*) prolonged by 34.69–43.32% for Bt-fed larvae, reaching 51.18 days for Ruifeng 125 second instars versus 35.71 days in controls (Table 1).

Both Bt events substantially suppressed growth parameters. The intrinsic rate of increase (*r_m_*) declined by 56.25–73.33%, with DBN9936 reducing third-instar *r_m_* to 0.06 (vs. 0.14 in Nonghua 106). Finite rates (λ) decreased by 8.55–10.34%, exemplified by Ruifeng 125-fed larvae (λ = 1.04–1.07 vs. 1.16–1.17 in controls). Population trend indices (*I*) collapsed by 95.16–98.50%, with DBN9936-fed third instars showing *I* = 6.89 versus 342.67 in non-Bt counterparts (Table 1).

*R*_0_-based relative fitness plummeted to 0.03–0.14 in Bt treatments, representing >85% reduction in population-level fitness. Third-instar larvae exposed to DBN9936 had a relative fitness of 0.09, while Ruifeng 125 yielded relative values ranging from 0.03 to 0.14. These data demonstrate that both Cry1Ab-expressing and Cry1Ab/Cry2Aj events impose substantial reductions in demographic fitness on *M. loreyi* populations (Table 1).

### 2.6. Larval Performance in M. loreyi Under Migration Simulation Scenarios

**Figure 5:**
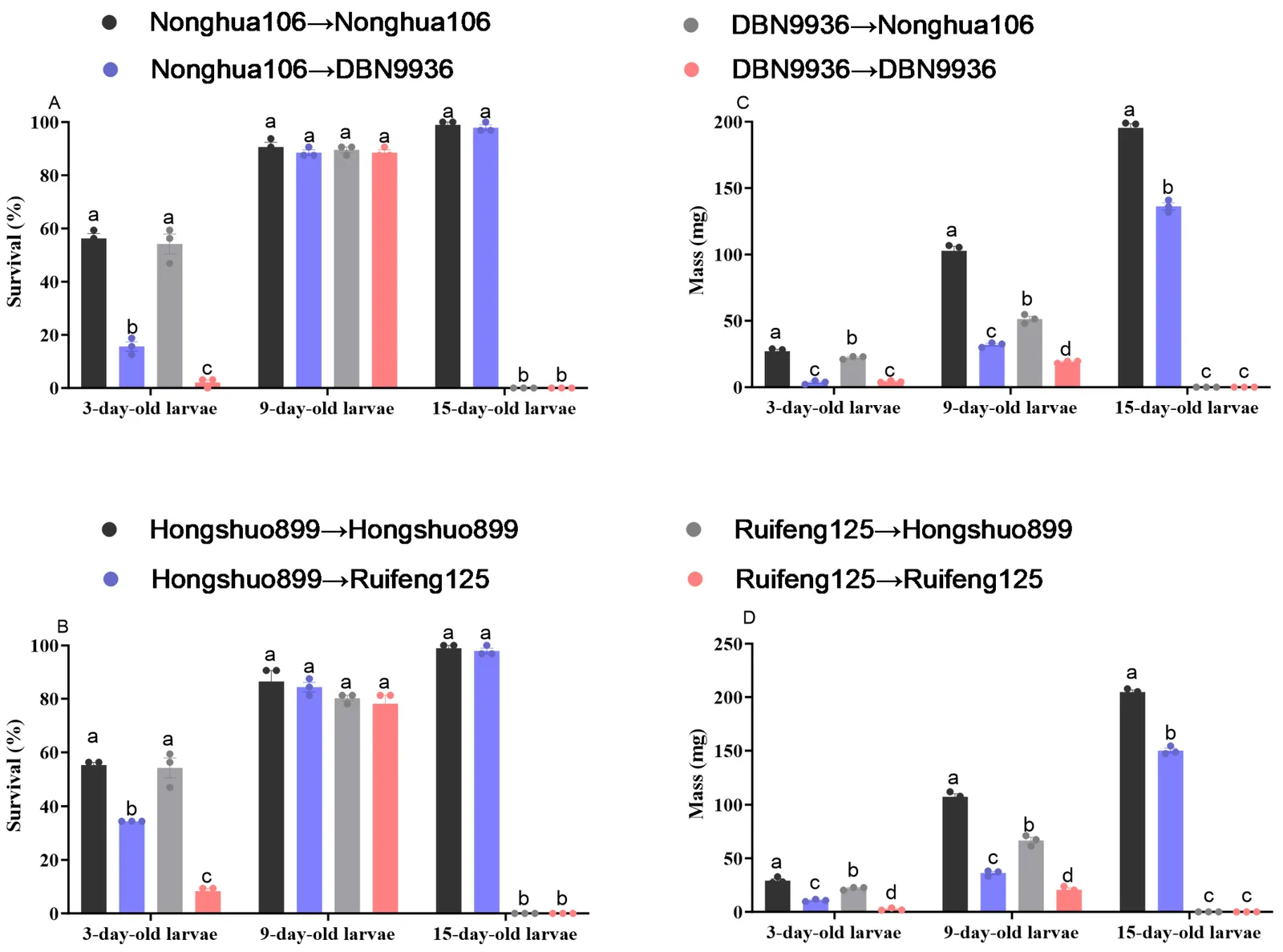
Survival and body mass of *M. loreyi* larvae (3, 9 and 15 days old), measured 7 days after transfer between leaves of different maize types. Bars represent mean values ± standard error (SE); Different lowercase letters denote significant differences among treatments (*p* < 0.05). Panels (**A**,**B**) were analyzed using generalized linear mixed models (GLMMs), whereas panels (**C**,**D**) were analyzed by Tukey’s HSD test. (**A**) Survival of larvae transferred between Nonghua 106 and DBN9936; (**B**) survival of larvae transferred between Hongshuo 899 and Ruifeng 125; (**C**) mass of larvae transferred between Nonghua 106 and DBN9936; (**D**) mass of larvae transferred between Hongshuo 899 and Ruifeng 125. No larvae survived to day 15 in the transgenic maize treatment.

Data for several transfer combinations were unavailable at 15 d, resulting in an incomplete factorial design. Consequently, the effects of transfer combination, time, and their interaction could not be tested independently, and each of the 20 observed combination–time treatments was analyzed as a separate factor level. Survival varied significantly among these treatments (χ^2^ = 426.95, df = 19, *p* < 0.0001; Figure 5A,B). Tukey-adjusted pairwise comparisons revealed significant differences among combinations at the 3 d time point, with the DBN9936→DBN9936 (2.08%) and Ruifeng→Ruifeng (8.33%) combinations showing lower survival (*p* < 0.001). However, no significant differences were detected among treatment combinations at 9 d and 15 d post-transfer, excluding groups where no individuals survived to 15 d on transgenic maize for subsequent testing (all *p* > 0.05). These results indicate that cultivar combination exerted significant effects on transfer-associated survival only during the early post-transfer phase (3 d); such effects were absent for individuals that had survived for 15 d on the initial maize cultivar.

Larval mass of *M. loreyi* after 7 days was significantly higher in scenarios involving continuous non-Bt feeding (Nonghua 106→Nonghua 106; Hongshuo 899→Hongshuo 899) or transfers from Bt to non-Bt maize (DBN9936→Nonghua 106; Ruifeng 125→Hongshuo 899), compared to Bt-exposed treatments (Figure 5C,D, *p* < 0.05). Consistently reduced body mass occurred when larvae were exposed to Bt maize, either through continuous feeding or post-transfer.

## 3. Discussion

This study systematically quantified the lethal and sublethal effects of two transformation events—DBN9936 (Cry1Ab) and Ruifeng 125 (Cry1Ab/Cry2Aj)—on early-instar larvae of *M. loreyi* under controlled conditions. DBN9936 induced 7-day corrected mortality of 93.43 ± 1.63% (first instar), 17.33 ± 4.05% (second instar), and 8.61 ± 0.67% (third instar) for *M. loreyi* (Figure 1A), while the corresponding mortality values for Ruifeng 125 were 44.33 ± 2.40%, 8.53 ± 1.33%, 3.34 ± 0.19%, respectively (Figure 1A). The sharp decline in mortality from first to third instars reveals a pronounced instar-dependent susceptibility of *M. loreyi* to Cry1Ab-based Bt maize. This instar-dependent susceptibility pattern is consistent with observations in other noctuid pests such as *S. frugiperda*, *O. nubilalis*, and *S. albicosta*, where late-instar larvae exhibit substantially higher survival on Bt maize or Bt toxin than neonates [23,24,25,26]. Notably, DBN9936 showed significantly higher control efficacy against neonate larvae compared to Ruifeng 125. This difference may be hypothetically associated with variations in Bt protein expression levels—the Cry1Ab protein content in DBN9936 during the V6-V8 leaf growth stage was 76.54 µg/g dry weight, approximately 1.87-fold higher than the total Cry1Ab/Cry2Aj expression of 40.86 µg/g dry weight in Ruifeng 125 [27,28].

Beyond direct lethal effects, Bt maize also induces significant sublethal effects—defined as physiological or behavioral fitness reduction in survivors following subtoxic exposure [29]. In this study, surviving larvae of *M. loreyi* that continued feeding on either DBN9936 (Cry1Ab) or Ruifeng 125 (Cry1Ab/Cry2Aj) exhibited prolonged larval development by 8.12–9.93 days, reduced pupation rates by 69.41–100.00% (0–27% across treatments), decreased pupal mass by 31.14–33.64%, and diminished fecundity by 177.86–262.96 eggs per female. This response pattern aligns with documented sublethal impacts in other Lepidopteran pests such as *H. zea*, *Mythimna unipuncta*, *O. nubilalis*, and *S. frugiperda* exposed to Cry1Ab maize [21,30,31,32,33,34,35,36,37,38], indicating conserved physiological stress responses to Bt toxins among lepidopteran pests.

Life table analyses confirmed that Bt maize profoundly disrupts the population dynamics of *M. loreyi*. First-instar larvae failed to survive on either Bt event. For second-instar larvae, DBN9936 remained completely lethal, while Ruifeng 125 allowed minimal survival (R_0_= 7.51, relative fitness = 0.03). For third-instar larvae, partial survival occurred on both Bt events (DBN9936: R_0_ = 21.68, relative fitness = 0.09; Ruifeng 125: R_0_ = 32.34, relative fitness = 0.14), with r_m_ values (0.06–0.07) significantly lower than those on conventional controls (0.14–0.16, all *p* < 0.001) (Table 1). These results carry several important implications. First, the near-complete reproductive failure (relative fitness ≤ 0.14) across all surviving cohorts demonstrates that, even when larvae survive the initial exposure, their contribution to the next generation is severely curtailed. This suggests that the cumulative population-level impact of non-high-dose Bt maize extends far beyond what mortality data alone would predict. Second, the stark contrast between complete lethality in first instars and partial survival in third instars reinforces the critical role of instar-dependent susceptibility in determining population outcomes—older larvae represent the primary demographic pathway for resistance allele persistence. Third, the extremely low r_m_ values (0.06–0.07) on both Bt events indicate that *M. loreyi* populations would require substantially more time to double under Bt selection pressure, potentially allowing natural enemies and other mortality factors to exert greater regulatory influence [34,39]. Collectively, these life table parameters underscore that, while neither Bt event meets the high-dose standard, their combined lethal and sublethal effects impose a severe demographic bottleneck that could suppress *M. loreyi* populations below economically damaging levels in low-pressure regions, albeit with the inherent risk of selecting for resistant individuals over successive generations.

Larvae migrating from non-Bt to Bt maize maintained the capacity to complete development, particularly older-aged larvae. Our study demonstrates that interplant movement under sublethal exposure to Bt protein promotes survival in this context. This behavioral pattern aligns with observations of *Busseola fusca* larval movement and survival within maize plantings with different ratios of non-Bt and Bt seed maize [40]. Such mobility-driven survival may accelerate resistance evolution in field populations of both species by maintaining allelic flow between exposed and refuge cohorts [41]. First, larval movement behavior functionally reduces the effective isolation between Bt and refuge plants—a cornerstone assumption of the high-dose/refuge strategy [20]. When larvae can move from non-Bt to Bt plants and survive, the intended genetic separation between susceptible (refuge) and potentially resistant (Bt-exposed) individuals is compromised, undermining the strategy’s ability to delay resistance. Second, this behavior creates a selective gradient across the landscape: neonates are strongly selected against in Bt plants, but older larvae that colonize Bt plants later in development face substantially weaker selection, allowing partially resistant genotypes to persist and reproduce. It is important to note that our experiments were conducted under controlled laboratory conditions simulating various migration scenarios. While these assays provide mechanistic evidence for movement-mediated survival, the extent to which *M. loreyi* engages in interplant movement between Bt and non-Bt maize under open-field conditions remains unknown. Factors such as plant spatial arrangement, wind direction, natural enemy presence, and alternative host availability may modulate movement behavior in the field. Therefore, field validation studies are critically needed to quantify the actual frequency and distance of interplant movement and to assess its real-world impacts on resistance evolution.

In summary, although the transformation events DBN9936 (Cry1Ab) and Ruifeng 125 (Cry1Ab/Cry2Aj) do not meet the “high-dose” standard against *M. loreyi*, their synergistic lethal and sublethal effects—including suppressed larval pupation, reduction in female adult fecundity, and delayed population growth—can contribute to sustainable management in areas with low infestation pressure. However, such “non-high-dose” traits carry inherent risks, as prolonged sublethal exposure may accelerate resistance evolution in surviving individuals. This concern is exemplified by the rapid field-evolved resistance of *S. frugiperda* to non-high-dose Bt traits [42]. To mitigate these risks, regionally tailored resistance management strategies are essential. These findings offer important implications for resistance management practice against *M. loreyi*. In regions with high *M. loreyi* pressure, multi-gene pyramided Bt maize may help improve enhance control efficacy and prolong the durability of Bt traits.

## 4. Conclusions

This study demonstrates that DBN9936 (Cry1Ab) and Ruifeng 125 (Cry1Ab/Cry2Aj) exert substantial lethal and sublethal effects on *M. loreyi*. Mortality was strongly instar-dependent. Survivors suffered severe fitness costs (relative fitness = 0.03–0.14), and population growth was significantly suppressed (r_m_ reduced by >50%). However, the ability of older larvae to survive on Bt maize, combined with potential interplant movement, creates a pathway for resistance allele persistence.

## 5. Materials and Methods

### 5.1. Transgenic Maize Events

The Cry1Ab protein-expressing maize event DBN9936 and its isogenic non-Bt control (Nonghua 106) were provided by Beijing Dabeinong Biotechnology Co., Ltd. (Beijing, China). The Cry1Ab/Cry2Aj fusion protein-expressing event Ruifeng 125 and its isogenic control (Hongshuo 899) were provided by Hangzhou Ruifeng Bioengineering Co., Ltd. (Hangzhou, China). Both transformation events were granted biosafety certificates by the Chinese government in 2019 [43]. All materials were cultivated under greenhouse conditions at the Institute of Plant Protection, Henan Academy of Agricultural Sciences (34.79° N, 113.67° E) (Zhengzhou, China). During the V6-V8 growth stages, the youngest fully expanded leaves (≥20 cm length) were excised from the plant tip. The presence of Bt protein content in leaf samples (which were from the same batch used in the larval bioassays) was quantitatively analyzed using commercial Cry1Ab/Ac-specific immunochromatographic rapid test strips (YouLong Biotechnology Co., Ltd., Shanghai, China) based on ELISA lateral flow assay technology.

### 5.2. Insect Colonies

*Mythimna loreyi* larvae were collected from the spring maize field in Ganan Town (32.12° N, 114.05° E), Pingqiao District, Xinyang City, Henan Province in May 2019. Colonies were maintained on an artificial diet consisting of corn flour, soybean meal, wheat germ, wheat bran, and casein [44]. Adults were housed in mesh cages (40 cm × 30 cm × 25 cm) provisioned with 10% (*v*/*v*) honey solution as a nutritional supplement. Oviposition substrates comprised nylon ropes. All insect stages were reared under controlled conditions: 27 ± 1 °C, 60 ± 10% RH, and a photoperiod of 16:8 h (L:D).

### 5.3. Effects of Transgenic Insect-Resistant Maize on Larval Development and Adult Reproduction in M. loreyi

Bioassays were conducted under controlled laboratory conditions (27 ± 1 °C, 60 ± 10% RH, 16:8 h L:D photoperiod). Young whorl leaves (V6-V8 growth stages) were collected from two pairs of maize plants. Leaves were cut into sections (approximately 1 cm × 2 cm) and placed into 32-well rearing plates (Frontier Agricultural Sciences, Newark, NJ, USA). Each well contained 3–4 leaf sections and 10 mL of 1% agar to maintain humidity. First-, second-, or third-instar larvae of *M. loreyi* were individually transferred into wells using a fine brush. Plates were sealed with breathable membranes to prevent larval escape. Fresh leaf sections were replaced daily. For each maize variety and larval instar, three independent biological replicates were performed, each consisting of 192 larvae (total *n* = 576 larvae per instar per cultivar). Larval survival and development duration were recorded daily. Pupation was monitored; pupae were weighed 2 days after pupation and sexed. Pupal duration was also recorded.

Newly eclosed adults (one female and one male) from each maize hybrid were paired in 500 mL rearing containers. The container tops were covered with gauze, and a nylon string was suspended inside each container to serve as an oviposition substrate. A plastic dish (2 cm diameter) containing absorbent cotton soaked in 10% sucrose solution was placed in each container for adult nutrition; the solution was replenished daily. All containers were maintained under controlled conditions: 27 ± 1 °C, 60 ± 10% RH, and a 16:8 h (L:D) photoperiod. Three replicates were conducted per maize hybrid, each replicate consisting of 10 mating pairs (*n* = 30 pairs per hybrid). Adult survival and oviposition were monitored daily until all adults died.

### 5.4. Effects of Host Plant Movement Scenarios on Development of M. loreyi

Movement of 3-, 9-, and 15-day-old *M. loreyi* larvae between non-Bt and transformation events was simulated under controlled conditions. Treatments represented distinct migration scenarios: larvae originating on Nonghua 106 (non-Bt) were transferred to either DBN9936 (Bt) or maintained on Nonghua 106 (control); larvae originating on DBN9936 (Bt) were transferred to either Nonghua 106 (non-Bt) or maintained on DBN9936 (control). Analogous treatments were implemented for larvae reared on Hongshuo 899 (non-Bt) and Ruifeng 125 (Bt); that is, transfers were conducted from Hongshuo 899 to Ruifeng 125 or Hongshuo 899 (control), and from Ruifeng 125 to Hongshuo 899 or Ruifeng 125 (control).

Bioassays were initiated by inoculating whorl leaves with first-instar *M. loreyi* larvae. Larvae fed for 3, 9, or 15 days before transfer onto V6-V8-stage leaves of the respective maize treatments. Surviving larvae were then individually placed into 32-well rearing plates, with each well containing fresh leaf tissue from the assigned treatment group. Plates were sealed with breathable membranes to prevent escape. Each treatment utilized 32 larvae per replicate, with three independent replicates (total *n* = 96 larvae per treatment). Leaf tissue was replaced every 2–3 days. All experiments were maintained at 27 ± 1 °C, 60 ± 10% RH, and a 16:8 h (L:D) photoperiod. Seven days post-transfer, larval survival rate and body weight (including surviving larvae and any pupae) were recorded.

### 5.5. Statistics and Analysis

Corrected mortality rates for *M. loreyi* on V6-V8-stage maize transformation events were calculated using Abbott’s formula [45]. Developmental duration of each life stage, larval survival rate, pupation rate, adult emergence rate, fecundity, and egg hatch rate were used to construct the age-specific life table and estimate population parameters [46,47]. The corresponding calculation formulas are presented as follows:

$$Larval\;survival\;rate\;(\%)=\frac{Number\;of\;surviving\;larvae\;at\;t2}{Number\;of\;larvae\;at\;t1}\times 100$$

$$Pupation\;rate\;(\%)=\frac{Number\;of\;pupated\;larvae}{Initial\;number\;of\;larvae}\times 100$$

$$Emergence\;rate\;(\%)=\frac{Number\;of\;emerged\;adults}{Initial\;number\;of\;larvae}\times 100$$

$$Egg\;hatch\;rate\;(\%)=\frac{Number\;of\;hatched\;larvae}{Total\;number\;of\;eggs}\times 100$$

Parameters included the net reproductive rate (*R*_0_ = ∑*l_x_m_x_*), mean generation time (*T =* ∑*xl_x_m_x_*/*R*_0_), intrinsic rate of increase (*r_m_* = ln*R*_0_/*T*), and finite rate of increase (*λ* = $e^{r_{m}}$), Where *x* denotes the age interval, *l_x_* is the proportion of females surviving to the start of age interval *x*, and *m_x_* is the mean number of female offspring produced per female during age interval *x*. Relative fitness was calculated as *R*_0_ (Bt)/*R*_0_ (non-Bt), where *R*_0_ represents the net reproductive rate [48]; t1 = initial time, t2 = end time.

All experimental data were organized using Microsoft Excel^®^ 2022. Statistical analyses were performed in R 4.5.3, and figures were generated with GraphPad Prism^®^ 9.5. Each treatment included three biological replicates, with 192 larvae examined per replicate, and replicate-level mean values were used for statistical analysis (*n* = 3). The Shapiro–Wilk and Levene’s tests were applied to check normality and homogeneity of variance prior to parametric tests.

Mortality, survival, pupation, emergence, percentage of female, and egg hatch rate data were analyzed using generalized linear mixed models (GLMMs). Binomial GLMMs were fitted for mortality, pupation, emergence and survival (dispersion test: *p* = 0.45, *p* = 0.21, *p* = 0.17, *p* = 0.41, respectively). Due to significant overdispersion, percentage of female and egg hatch rate data were fitted with a beta-binomial GLMM. Model selection was based on the Akaike information criterion (AIC).

Two-way ANOVA was used to evaluate the effects of maize variety (fixed factor), larval instar (fixed factor), and their interaction on the biological parameters of *M. loreyi*. One-way ANOVA, followed by Tukey’s HSD test or independent *t*-tests (α = 0.05), compared larval body weight and life history parameters among varieties within each instar and between Bt and non-Bt maize.

Life table parameters (*R*_0_, *T*, *r_m_*, *λ*) were calculated in Microsoft Excel 2022 using traditional age-specific life tables from group-level survival and fecundity data. Dead and missing individuals were recorded at group level. Individual-level raw data were unavailable under group rearing; thus, bootstrap resampling was not implemented, and standard errors and confidence intervals for life table parameters were not estimated. Life table parameters were used only for descriptive comparison, without significance testing, and were not treated as normally distributed data for inference.

## Figures and Tables

**Table 1:** Life table parameters of *M. loreyi* fed on different maize varieties under controlled conditions.

Larvae	Corn Varieties	Net Reproductive Rate (*R*_0_)	Mean Generation Period (*T*)	Intrinsic Rate of Increase (*r_m_*)	Finite Rate of Increase (*λ*)	Population Growth Trend Index (*I*)	Relative Fitness (*R*)
1st instar	Nonghua106	165.73	39.43	0.13	1.12	311.15	1
	DBN9936	/	/	/	/	/	/
	Hongshuo899	215.29	37.13	0.14	1.14	423.83	1
	Ruifeng 125	/	/	/	/	/	/
2nd instar	Nonghua106	174.20	37.86	0.13	1.15	319.12	1
	DBN9936	/	/	/	/	/	/
	Hongshuo899	219.22	35.71	0.15	1.16	447.08	1
	Ruifeng 125	7.51	51.18	0.04	1.04	6.72	0.03
3rd instar	Nonghua106	245.51	36.90	0.14	1.16	342.67	1
	DBN9936	21.68	49.70	0.06	1.06	6.89	0.09
	Hongshuo899	232.59	35.12	0.16	1.17	445.28	1
	Ruifeng 125	32.34	48.43	0.07	1.07	21.57	0.14

“/” indicates that no individuals survived on the corresponding maize variety at that instar, precluding parameter estimation.

## Data Availability

The original contributions presented in this study are included in the article. Further inquiries can be directed to the corresponding author.
